# COVID‐19 and cardiac surgery: Do outcomes differ?

**DOI:** 10.1111/jocs.14977

**Published:** 2020-08-26

**Authors:** Amer Harky, Grace Poole, Ariana Axiaq, Bilal H. Kirmani

**Affiliations:** ^1^ Department of Integrative Biology, Faculty of Life Sciences University of Liverpool Liverpool UK; ^2^ Department of Cardiothoracic Surgery Liverpool Heart and Chest Hospital Liverpool UK; ^3^ St George's School of Medicine University of London London UK; ^4^ School of Medicine Queen's University Belfast Belfast UK

**Keywords:** cardiac surgery, COVID‐19, healthcare provisions, LMIC, SARS‐CoV‐2

Coronavirus disease 2019 (COVID‐19) has had an unprecedented impact on the provision of healthcare services worldwide. The novel severe acute respiratory syndrome coronavirus 2 (SARS‐CoV‐2) was first declared by the World Health Organization as a global pandemic on 11th March 2020.^1^ To date COVID‐19 has affected over 190 countries, with over 19 million confirmed cases and 710,000 associated deaths,^2^ vastly increasing the global strain on healthcare services and significantly impacting all elective surgeries. The natural history of cardiac surgical disease, however, has required judicious management of these high‐risk patients. Given the reliance of cardiac surgery on extensive peri‐ and postoperative resources including anesthetic and intensive care staff, blood products and ventilators; the need to triage and manage resources, when these facilities are scarce, is imperative.^3^, ^4^ This has created a global necessity for cardiac surgery programs to adapt during such pandemic.

While a wealth of research has been published evaluating adaptations within the developed countries, comparatively little has been published outlining the provision of services in developing countries.^4^, ^5^, ^6^ These shortcomings are highly significant given that, in such countries, barriers to surgical care and disease burden vary greatly in comparison.^7^, ^8^ Ramsingh et al reported their experience of 58 patients who had undergone cardiac surgery in two centers between April and June 2020. Three of those patients were acute aortic dissections while the rest were combination of urgent coronary artery bypass grafting and/or symptomatic aortic valve replacements. Patient selection was identified through multidisciplinary team (MDT) discussions and surgery planned accordingly.^9^ Operations on these patients was carried out using personal protective equipment (PPE) and minimising theatre staff to the lowest safe possible numbers. Their experience reflects how cardiac surgery has been adapted safely in the high‐income developing country of Trinidad and Tobago (T&T) during these unprecedented times.^9^ While T&T have the second highest income per capita in the region, national health indicators significantly lag behind economic growth and substantial pockets of poverty remain.^10^ Despite its small population of 1.40 million, T&T has the world's 34th highest population density, similar to that of the United States of America. It has had, nonetheless, remarkably low COVID‐19 cases (142 cases per one million population, perhaps reflecting a low testing rate) and low associated deaths (eight deaths in total).The twin island state therefore provides a unique opportunity to examine successful strategies to safely deliver cardiac surgery in a developing country.^11^, ^12^ These changes may subsequently be compared with low‐ and middle‐income countries (LMICs), in addition to their developed counterparts.

Like many countries across the globe, the burden on healthcare services resulted in the suspension of all elective surgeries in T&T.^9^, ^13^ Their deferral proves to be an important step in enabling the reallocation of limited staff to intensive care units (ICUs) and rationing the use of supplies including PPE and blood products. The introduction of aggressive healthcare policies was deemed essential to reduce the risk of transmission and likelihood of patients acquiring nosocomial COVID‐19 infection. Such precautions stem from the fact that patients with pre‐existing cardiovascular disease are more likely to have unfavorable clinical outcomes with COVID‐19 infection.^14^ In T&T, these aggressive policies took the form of a mass restructuring of healthcare into “parallel systems” with “hot” (COVID‐19 presumed or proven) and “cold” (COVID‐19 negative) centers.^9^ While such a strategy has been possible in high‐income countries, limited accessibility and border closures in LMICs meant many were forced to rely on a single center to cope with a surge in COVID‐19 infections. The redistribution of resources within these centers reduced cardiac surgical services to a bare minimum.^15^ This was exacerbated by the disruption of conventional supply chains and surgical donations to LMICs following border closures and travel restrictions.^7^

Within developed countries, health demographics and COVID‐19 related burden were also key influencers of resource management and allocation. In National Health Service (NHS) England, a protocol known as Pan‐London Emergency Cardiac surgery (PLECS) was designed to permit cardiac surgery for urgent and emergency cases. From a total of seven cardiac centers based in London, two were selected as central hubs, with access to their Accident and Emergency departments suspended to maintain a COVID‐19 free environment.^16^ Healthcare systems within the United States of America adopted a similar approach.^17^ Meanwhile, in Canada, a three‐stage system with a variable degree of service reduction was implemented.^18^ In developed countries with extensive viral spread, a “hub‐and‐spoke” model was preferred for delivering cardiac surgery. This entailed a select number of tertiary centers being assigned as restricted‐access cold operating centers (Hubs), and a majority of other cardiovascular centers were open‐access hot referring units with surgery suspended, termed spokes. Hubs focused on the provision of emergency cardiac surgery, whilst spokes concentrated on patient enrollment.^19^ This strategy was convenient to re‐distribute critical resources for COVID‐19 patients.

Canceling elective surgery has also provided a new challenge for a multitude of cardiac surgical teams. There has been a need to differentiate between patients requiring urgent care and those for whom surgery can be safely postponed.^3^ Risks of deferring surgery include worsening of the patient's condition, which may subsequently compromise their fitness for later procedures. To make this distinction, international cardiac services were required to triage and prioritise their patients. A notable difference between T&T and other HICs, was the use of telemedicine. An important aspect of telehealth in cardiac surgery was tele‐triage, allowing for the risk stratification of patients.^20^ Within PLECS, patients were categorised into four levels based on their urgency. Elective patients were classified as level 1; level 2 consisted of patients at home in need of urgent care; inpatients occupied level 3 and level 4 were emergency cases requiring urgent surgery.^16^ While telecardiology has been recommended by the American College of Surgery, T&T continued to triage utilising MDT (cardiac surgical teams) in preoperative clinics, with enforced social distancing measures.^9^ Assuming that the 58 patients operated on during the two‐month study period were distributed evenly, this is a manageable footfall for a department, however this model is unlikely to have been possible in services with larger caseloads.

In addition to difficulties with surgical triage, there is concern over the backlog of cases that canceling or postponing elective surgical procedures has created. There is an estimated worldwide backlog of nearly 30 million elective procedures in just 12 weeks.^13^ In LMICs, where surgical delivery is already constrained, the pandemic may have even greater consequences.^8^ The scale of the repercussions has been felt globally with even well‐resourced surgical healthcare systems, such as the NHS in UK and North American health care systems struggling to cope with the ever‐increasing demands of this backlog.

There are some parallels between the management of patients requiring cardiac surgery in developed and developing countries. Measures, such as social distancing, use of PPE, enhanced precautions for aerosol generating procedures (AGPs) and quarantine have been universally adopted. Other measures, such as reverse transcriptase polymerase chain reaction (RT‐PCR) testing of prospective patients have been resource dependent.^22^ T&T have performed 9390 COVID tests during this pandemic. By comparison a third of this number were performed by the authors' (AAH/BHK) single tertiary level hospital in the United Kingdom. The extent of the availability of this testing depends on many factors, including access to financial resources within healthcare services. While private hospitals in T&T are able to provide preoperative RT‐PCR testing for all patients, this is not the case for public healthcare services. A limited availability of tests resulted in only symptomatic cardiac surgical patients received preoperative RT‐PCR.^9^ Given that 43% to 60% of patients with COVID‐19 are thought to be asymptomatic, this has implications for risk stratification and preventing disease spread.^3^, ^23^, ^24^ Therefore, testing for SARS‐CoV‐2 in patients undergoing cardiac surgery is important as many of such patients are asymptomatic and may have subclinical infections which could have significant impact on peri‐operative outcomes.^25^

An international cohort study, including 1128 patients, organised by COVIDSurg Collaborative, report a 23.8% postoperative 30‐day mortality (n = 268) in patients infected with SARS‐CoV‐2.^26^ In addition to an increase in overall mortality, the use of cardiopulmonary bypass (CPB) in patients with COVID‐19 is thought to increase the risk of acute respiratory distress syndrome (ARDS). It is hypothesised that exposure of blood to non‐endothelial surfaces during CPB triggers a proinflammatory response, increasing both tumor necrosis factor α and interleukin 10,^16^ two cytokines implicated in ARDS in COVID‐19.^3^, ^27^

Given the associated risks and frequency of AGPs involved in cardiac surgery, preoperative testing plays a significant role in resource allocations. Thus, in developing countries, where testing may be restricted, the authors demonstrate the two different approaches to dealing with this conundrum. In the private sector, they demonstrated the use of prioritising tests to high risk groups (those undergoing cardiac surgery) and in the state hospitals ensuring that all other preventative measures were executed rigorously. One possible method to do so is by implementing a preoperative testing algorithm, such as one recommended by Patel et al,^3^ based on patients' travel, exposure history and community prevalence. This is perhaps a more reliable method than symptom manifestation. Such testing mechanism should be complemented by strict preoperative shielding measures, the likes of which have already been implemented within T&T.^9^

Surgical equipment and SARS‐CoV‐2 testing have not been the only global scarcity during this pandemic. Blood transfusions have also been restricted due to a limited number of blood donations, secondary to national and regional lockdowns. Such issues have been reported by Ramsingh et al to have particularly affected T&T causing a national shortage.^9^ They were able to mitigate for this by utilising off‐pump coronary surgery and use of autologous blood transfusion.

It is not only limitations of tangible resources, including ventilators and PPE, that provide restrictions on the provision of cardiac surgery. During COVID‐19, many healthcare systems reallocated surgical staff to the emergency frontline and ICUs to overcome the burden of the pandemic.^4^, ^8^, ^29^ Although Ramsingh et al reported that many countries, such as UK, Italy, and Canada have practiced the redeployment of cardiac surgical staff to general critical care areas to facilitate ventilated patients with COVID, their own staff redeployment remains unclear. The mandatory 2‐week isolation of cardiac surgical staff outside of work was an additional precaution made in T&T that was particularly patient‐centric. Such requirements of healthcare workers would probably not be feasible in developed countries but were implemented in T&T to compensate for the reduced levels of testing. Before the pandemic, the Lancet Commission on Global Surgery estimated a need of 20 surgeons, anaesthesiologists and obstetricians per 100,000 population for LMICs to meet the burden of surgical disease. Yet, statistics show LMIC only met half of this recommendation.^30^ This shortage may only be compounded by deficiencies in PPE, staff burn‐out and inevitable exposure of health care workers to SARS‐CoV‐2.^8^

The impact of COVID‐19 has been felt throughout healthcare systems across the globe, indiscriminately affecting developed and developing nations.^31^ In many specialties including cardiac surgery, caution and improvisation has been essential to continue provide services to those in whom the risks of the natural history of their disease outweigh those of the risks of catching COVID‐19 in hospital.^32^ This is particularly true of cardiac surgical disease. Between the 190 countries that have been affected by COVID‐19, each nation's accessibility to such resources vary widely. Ramsingh et al demonstrate well that basic measures, such as social distancing, mandatory preoperative isolation and rigorous PPE can supplement and mitigate for reduced access to swab testing. They demonstrate well that there is no universally applicable worldwide framework for the provision of cardiac surgery during the pandemic. Individual nations' responses to the pandemic must consider their service burden and availability of resources, including staff and undertake appropriate surgical prioritization, triage and preoperative assessment. It appears to be possible to balance the risks of peri‐operative COVID infection with those of advanced cardiac pathology.^33^
